# Cytotaxonomic study of the Chilean endemic complex *Alstroemeria magnifica* Herb. (Alstroemeriaceae)

**DOI:** 10.1590/1678-4685-GMB-2017-0157

**Published:** 2018-05-14

**Authors:** Carlos M. Baeza, Víctor Finot, Eduardo Ruiz, Pedro Carrasco, Patricio Novoa, Marcelo Rosas, Oscar Toro-Núñez

**Affiliations:** 1 Universidad de Concepción Universidad de Concepción Facultad de Ciencias Naturales y Oceanográficas Departamento de Botánica Concepción Chile Departamento de Botánica, Facultad de Ciencias Naturales y Oceanográficas, Universidad de Concepción, Concepción, Chile; 2 Universidad de Concepción Universidad de Concepción Facultad de Agronomía Departamento de Producción Animal Chillán Chile Departamento de Producción Animal, Facultad de Agronomía, Universidad de Concepción, Chillán, Chile; 3 Jardín Botánico Jardín Botánico Viña del Mar Chile Jardín Botánico, Viña del Mar, Chile; 4 Universidad de Concepción Universidad de Concepción Departamento de Botánica Programa de Doctorado en Sistemática y Biodiversidad Concepción Chile Programa de Doctorado en Sistemática y Biodiversidad, Departamento de Botánica, Universidad de Concepción, Concepción, Chile

**Keywords:** Alstroemeria, karyotype, species complex, asymmetry, Chile

## Abstract

*Alstroemeria* L. (Alstroemeriaceae) represents one of the most diverse genera of vascular plants in Chile. It contains approximately 54 taxa, 40 of which are endemic. The “complex” *Alstroemeria magnifica* is endemic to Chile, and it comprises four varieties: *A. magnifica* var. *magenta*, *A. magnifica* var. *magnifica*, *A. magnifica* var. *sierrae*, and *A*. *magnifica* var. *tofoensis*. It is distributed from Coquimbo to the Valparaíso Region. We analyzed karyotypes of 10 populations along its natural distribution. All the populations presented an asymmetric karyotype, with 2*n* = 16 chromosomes but with three different karyotypic formulae. *Alstroemeria magnifica* var. *magnifica* and *A. magnifica* var. *sierrae* presented the same karyotypic fomula, and *A.* magnifica var. *magenta*, and *A. magnifica* var. *tofoensis* each had a different formula. The scatter plot among CV_CL_ *vs*. M_CA_ shows different groupings between populations of the four varieties. Based on the results, it is possible to consider raising *Alstroemeria magnifica* var. *magenta* to species level (*A*. *magenta*) and *A. magnifica* var. *tofoensis* to subspecies level (*A. magnifica* subsp. *tofoensis*); *A. magnifica* var. *magnifica* and *A. magnifica* var. *sierrae* should each remain as varieties. Nevertheless, these taxonomic changes should be considered tentative, as additional sources of evidence become available.

## Introduction

*Alstroemeria* is a South American genus, which comprises about 82 taxa distributed from Venezuela (3°N) to Tierra del Fuego (53°S) (Muñoz and Moreira, 2003). The centers of distribution of this genus are located in Central Chile and East of Brazil, representing a disjoint pattern of distribution produced by the isolating effect of the Cordillera de Los Andes and the South American Arid Diagonal (Muñoz and Moreira, 2003; Hofreiter, 2007; Chacón *et al.*, 2012a).

In Chile, *Alstroemeria* represents one of the most diverse genera of vascular plants, comprising 49 taxa (33 species, 8 varieties, and 8 subspecies); 40 of which are endemic (Muñoz and Moreira, 2003). Recent studies suggest increasing to 54 the number of taxa recognized in *Alstroemeria*, with the validation of *Alstroemeria citrina* Phil. (Eyzaguirre, 2008) and *Alstroemeria parvula* Phil. (Muñoz *et al.*, 2011). These modifications also include the discovery of *Alstroemeria hookeri* Lodd. subsp. *sansebastiana* C.M. Baeza & E. Ruiz (Baeza and Ruiz, 2011), *Alstroemeria marticorenae* Negritto & C. M. Baeza (Negritto *et al.*, 2015) and *Alstroemeria traudliae* (Hoffmann *et al.*, 2015).

Reports of chromosome studies in *Alstroemeria* are dated since 1882, recognizing a fundamental karyotype on about 30 taxa, 22 of them from Chilean species (Chacón *et al.*, 2012b). A stable chromosome set of 2*n* = 16 was determined, with an asymmetric and bimodal karyotype of eight chromosomes: three or four are acrocentric and four or five are metacentric, submetacentric or subtelocentric (Baeza *et al.*, 2008). Until today, no reports of polyploids have been observed in natural populations of *Alstroemeria* (Baeza *et al.*, 2007a).

Cytogenetic studies have proven useful for the delimitation of entities in *Alstroemeria* since every studied taxon presents a distinctive, unique, and largely stable karyotype. As such, these studies have contributed not only to the delimitation of species and varieties, but also to elicit underlying processes - at chromosomal levels - that determine the divergence of these taxa (Baeza *et al.*, 2007b). In taxonomic complexes, a clear-cut discrimination of intraspecific taxa has resulted from the study of differences in the architecture and/or the asymmetry of chromosomes (Cajas *et al.*, 2009; Baeza *et al.*, 2010). For example, the study of karyotype was determinant for the delimitation of taxa within the *Alstroemeria hookeri* complex, providing evidence for the existence of *Alstroemeria hookeri* subsp. *sansebastiana* (Baeza and Ruiz, 2011) and supporting the proposal of Muñoz and Moreira (2003) to raise the status of *Alstroemeria hookeri* subsp. *cummingiana* to species level.

The difficult differentiation and delimitation of taxa within *Alstroemeria* have led to the definition of intraspecific complexes, which consist of two or four subspecies and/or varieties of the same species. Some of these complexes have become relevant taxa for the prospection of development in different areas of national interest, given their economic potential as ornamental plants, and/or their importance as representative taxa of the Chilean biodiversity. Traditional taxonomic treatments in *Alstroemeria* have largely been based on patterns of variability of their conspicuous flowers, which present tepals with a wide display of morphological and coloring patterns (Bayer, 1987; Muñoz and Moreira, 2003). These structures, while useful for discrimination at interspecific levels, usually exhibit levels of variation beyond levels of stability required for a robust taxonomic discrimination in several groups of species (Baeza *et al.*, 2010, 2015, 2016a,b). Therefore, since any potential development in these groups has been mostly restricted to inconclusive taxonomic interpretations the use of other possible sources of evidence, like cytological characters, could result useful to help taxonomic discrimination at intraspecific levels.

**Figure 1:**
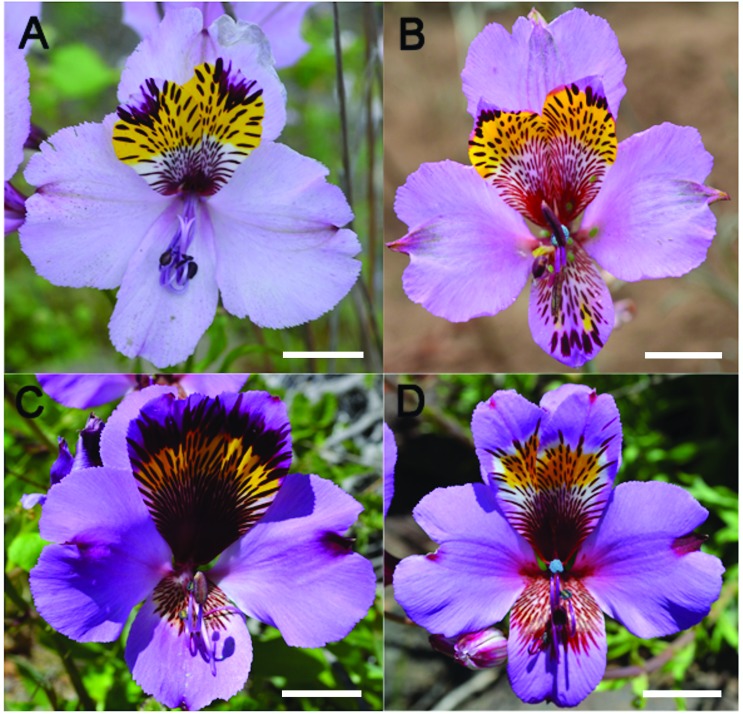
Photographic representation of varieties present in the *A. magnifica* complex. (A) Photography of *A. magnifica* var. *magnifica*; (B) Photography of *A. magnifica* var. *magenta*; (C) Photography of *A. magnifica* var. *sierrae;* (D) Photography of *A. magnifica* var. *tofoensis*. Bar = 2 cm.

*Alstroemeria magnifica* Herb. is a species complex which comprises four varieties (Muñoz, 2003; Muñoz and Moreira, 2003). Along with *A. hookeri*, *A. magnifica* is one of the richest in terms of number of taxa in *Alstroemeria*. This complex is distributed from the locality of Chungungo (29°26’S; Region of Coquimbo) to the north of Papudo (32°21’S; Region of Valparaiso). It is recurrent across coastal rocky bluffs and slopes, most likely in areas with permanent fog. The specific ranges of distribution and description of floral morphology are the following:

*Alstroemeria magnifica* Herb. var. *magnifica*: Distributed from 29º30’S to 30º 50’S. It is recognized by their whitish to lilaceous flowers, with a plain internal tepal (no design present; Figure 1A).

*Alstroemeria magnifica* Herb. var. *magenta* (Ehr.Bayer) Muñoz-Schick: This variety presents the largest range of distribution within the complex, ranging from 30°39’S to 32°21’S. It is distinguished by the presence of both small inflorescences and flowers. Their internal upper tepals show thick lines, which end in a respective spot at the apex. The internal lower tepal can present design or not (Figure 1B).

**Figure 2:**
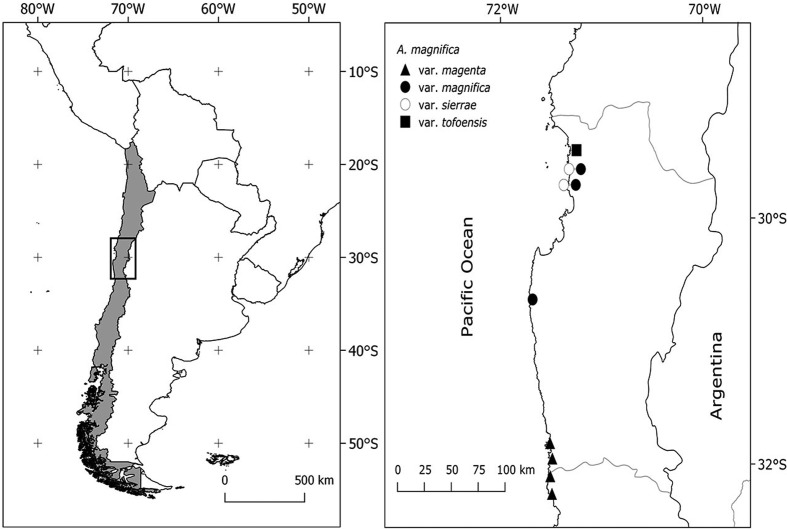
Geographic distribution of the 10 analyzed populations of *A. magnifica* complex.

*Alstroemeria magnifica* Herb. var. *sierrae* (Muñoz) Muñoz-Schick: This variety occurs in a restricted distribution, from 29º36’S to 29°45’S. It presents flowers of large size, which are distinctive by the design present in the internal upper tepals, and having lines forming a large spot at the apex and basis of this structure. The internal lower tepal can present design (Figure 1C).

*Alstroemeria magnifica* Herb. var. *tofoensis* Muñoz-Schick: This variety has a very restricted distribution, from 29°26’S to 29°32’S. It is characterized by their internal upper tepals with a patch of a yellowish spot and a white background, which does not reach the borders. Scattered lines with no spotty end at the apex are also present. The internal inferior tepal is maculate at the basis (Figure 1D).

Until now, cytological work has not been extensive for completely clarifying the taxonomic status of the infraspecific taxa of the *A. magnifica* complex. Buitendijk and Ramanna (1996) analyzed the karyotype of *A. magnifica* subsp. *magnifica*, informing the morphology of chromosomes and interspecific variability in the C-bands patterns. Additional reports exist about the genomic size (Buitendijk *et al.*, 1997, 1998), which in association with patterns of variation in C-bands, suggest discontinuous variation in the quantity of nuclear DNA of *A. magnifica*. Nevertheless, since these observations are circumscribed to cultivated specimens only (mostly cultivars and greenhouse varieties), no additional cytological information exists from local populations and/or range of variation within their natural environmental and geographic ranges.

Therefore, the present study aims to characterize and compare, at a cytotaxonomic level, the four varieties of the *A. magnifica* complex. Thus, using representative sampling from the total of the geographic range of distribution, we expect to offer a suggested clarification of the taxonomic status of each variety within the complex.

## Materials and Methods

### Plant material

A total of two to four individuals from 10 populations of *A. magnifica* were collected across the known range of distribution (Table 1). Voucher specimens from each population were deposited in the Herbarium of the University of Concepción (CONC). Figure 2 shows the distribution of the collected populations, which were used in the present study.

**Table 1:** Plant material for the analyzed populations.

Species	Population	Locality and Date of Collection.	Latitude S/ Longitudes W	Altitude (m)
*A. magnifica* var. *magnifica*	4408	Región de Coquimbo. Provincia de Elqui. Inicio Cuesta Buenos Aires. 5 de octubre de 2014	29º34’18’’/71º14’35’’	473
	4411	Región de Coquimbo. Provincia de Elqui. Entre Cuesta Porotitos y Caleta Hornos. 7 de octubre de 2014	29º44’32’’/71º19’20’’	150
	4414	Región de Coquimbo. Provincia de Limarí. Bosque Hidrófilo, parte alta. 8 de octubre de 2014	30º39’45’’/71º40’57’’	598
*A. magnifica* var. *magenta*	4379	Región de Coquimbo. Provincia de Choapa. Bosque Santa Julia, fundo Agua Amarilla. 31 0ctubre de 2013	31º49’48’’/71º30’35’’	110
	4380	Región de Coquimbo. Provincia de Choapa. Entre quebrada El Negro y Los Vilos. 31 octubre 2013	31º57’20’’/71º29’14’’	138
	4381	Región de Coquimbo. Provincia de Choapa. Fundo Palo Colorado, 5 km al norte de Puente Quilimarí, frente al Cerro Tentén. 1 de noviembre de 2013	32º05’58’’/71º30’27’’	80
	4383	Región de Valparaíso. Provincia de Petorca. 2 km al sur de Los Molles. 1 de noviembre de 2013	32º14’35’’/71º29’27’’	37
*A. magnifica* var. *sierrae*	4406	Región de Coquimbo. Provincia de Elqui. Juan Soldado. 5 de octubre de 2014	29º43’04’’/71º18’25’’	175
	4407	Región de Coquimbo. Provincia de Elqui. Caleta Hornos. 5 de octubre de 2014	29º38’01’’/71º17’08’’	152
*A. magnifica* var. *tofoensis*	4409	Región de Coquimbo. Provincia de Elqui. Mina El Tofo. 6 de octubre de 2014	29º26’56’’/71º14’52’’	676

### Methodology for the study of karyotypes

Rhizome roots (1-2 cm length) obtained from individuals in each population and held in a greenhouse, were cut and pre-treated with a solution of 8-hydroxyquinoline (2 mM) for 24 h at 4 ºC. These samples were subsequently fixed with a fresh solution of ethanol/acetic acid (3:1) for 24 h. Squash preparations from root tips were made using an acid hydrolysis pretreatment with HCL 0.5 N during 17 min at 42 ºC. After washing in distilled water, the material was stained with 1% orcein solution. Metaphase chromosome plates were photographed using a Zeiss Axioskop hmicroscope, with an incorporated video camera. Chromosomes were measured with the assistance of the software MicroMeasure 3.3 (Reeves, 2001) and classified according to arm ratios (long arm/short arm; modified from Levan *et al.*, 1964). From 10 metaphase plates in each analyzed population, randomly chosen from the total of individuals, an idiogram was constructed for each studied variety. Intrachromosomal asymmetry (M_CA_) and interchromosomal asymmetry (CV_CL_) indices were calculated for each analyzed population, following the proposal of Peruzzi and Erôglu (2013). Both indices were placed in a scatter plot, accompanied by the total length of diploid chromosomes (TCL), for each analyzed population using the package plot3D v 1.1 (Soetaert, 2016) in R v 3.3.3 (R Core Team, 2017).

## Results and Discussion

**Figure 3:**
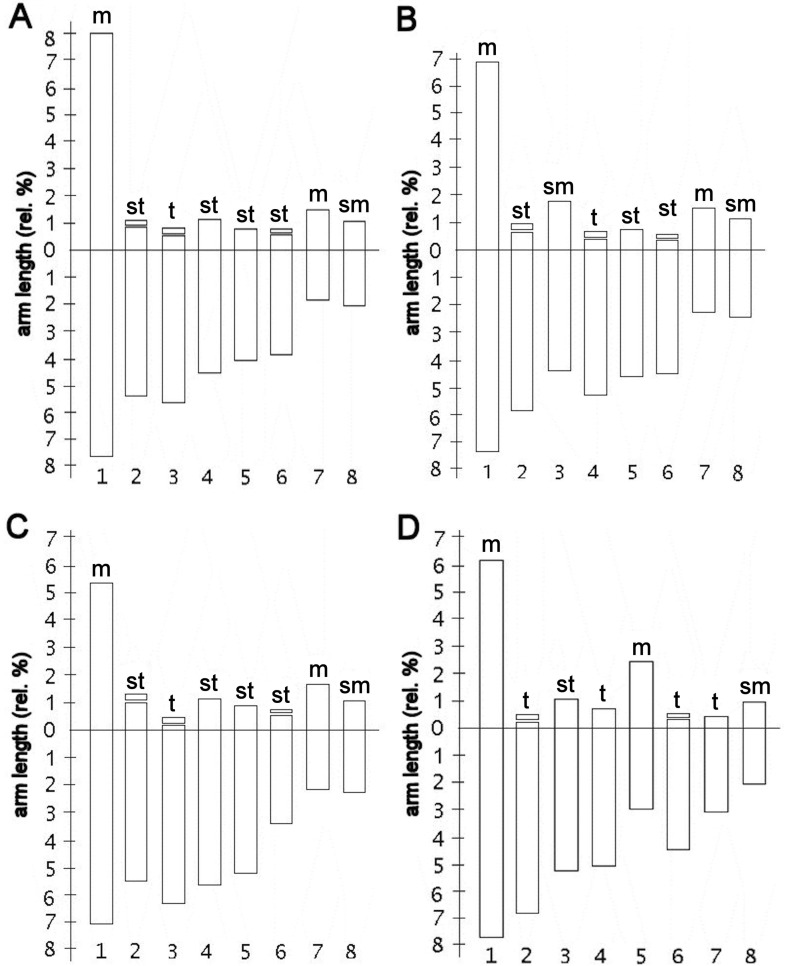
Idiograms of *A. magnifica* varieties. (A) *A. magnifica* var*. magnifica*; (B) *A. magnifica* var. *magenta*; (C) *A. magnifica* var. *sierrae*; (D) *A*. *magnifica* var. *tofoensis*.

**Figure 4:**
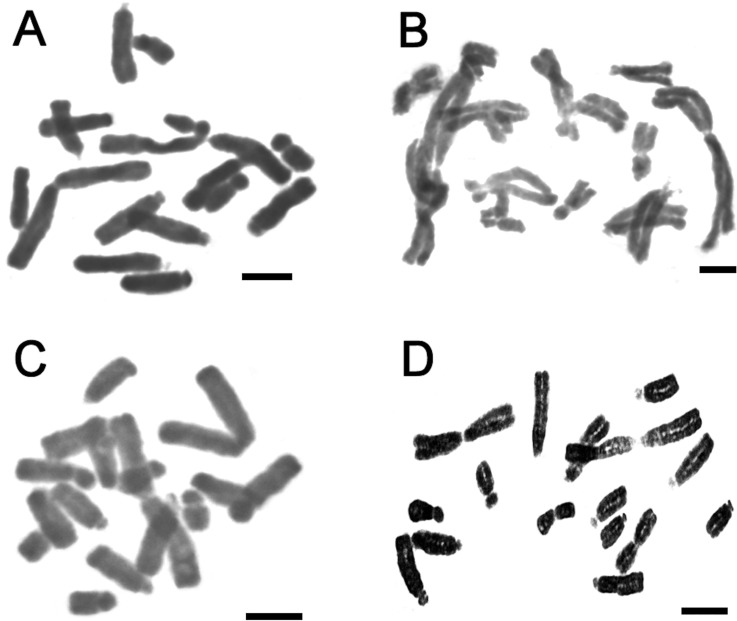
Metaphase plates in the varieties of *A. magnifica* complex. (A) *A. magnifica* var*. magnifica* (4408); (B) *A. magnifica* var. *sierrae* (4406); (C) *A. magnifica* var. *magenta* (4381); (D) *A*. *magnifica* var. *tofoensis* (4409)*.* Bar = 5 μm.

**Figure 5:**
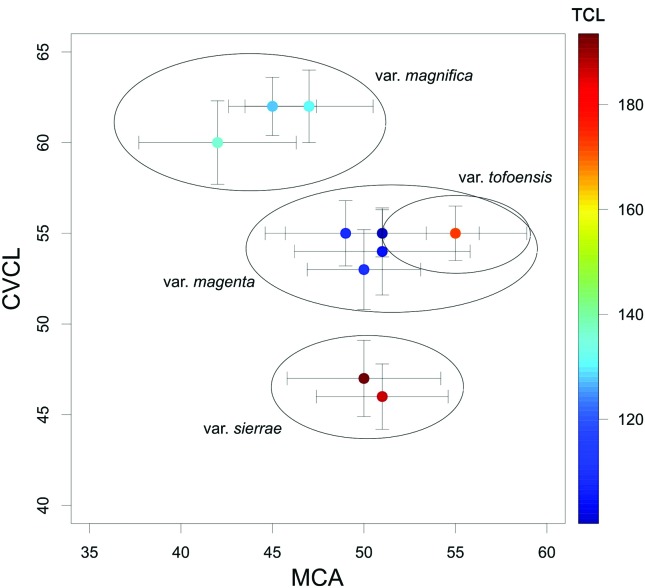
Scatter plot among populations of *Alstroemeria magnifica* varieties using values of M_CA_ *vs*. CV_CL_.

All analyzed populations of *A. magnifica* revealed a 2*n* = 2*x* = 16. *A. magnifica* var. *magnifica* and *A. magnifica* var. *sierrae* presented the same haploid formula: two pairs of metacentric chromosomes, one submetacentric pair, two subtelocentric pairs, two subtelocentric pairs with satellite and one telocentric pair with satellite (2m + 1sm + 2st + 2st-sat + 1t-sat; Figure 3A,C). A. *magnifica* var. *magenta* presented a haploid formula of two metacentric chromosomes, two submetacentric pairs, one subtelocentric pair, two subtelocentric pairs with satellite and one telocentric pair with satellite (2m + 2sm +1st + 2st-sat + 1t-sat; Figure 3B). *A. magnifica* var. *tofoensis* presented a haploid formula of two metacentric chromosomes, one submetacentric pair, one subtelocentric pair, two telocentric pairs and two telocentric pairs with satellite (2m + 1sm + 1st + 2t + 2t-sat; Figure 3D). Figure 4 shows representative metaphase plates for each studied taxon. The values of CV_CL_, M_CA_ and TCL per populations are summarized in Table 2. Figure 5 represents the scatter plot of CV_CL_ and M_CA_ indices.

**Table 2:** Karyotype features of the varieties of *Alstroemeria magnifica*. CV_CL_ = Coefficient of variation of chromosome length; M_CA_ = Mean centromeric asymmetry index according to Peruzzi and Erôglu (2013); SD = Standard deviation; TLC = Total length in diploid chromosomes.

	CV_CL_ ± SD	M_CA_ ± SD	TLC ± SD
*Alstroemeria magnifica* var. *magnifica* (4408)	62.0 ± 2.4	45.0 ± 1.6	128.2 ± 6.2
*Alstroemeria magnifica* var. *magnifica* (4411)	60.0 ± 4.3	42.0 ± 2.3	136.5 ± 8.3
*Alstroemeria magnifica* var. *magnifica* (4414)	62.0 ± 3.5	47.0 ± 2.0	130.4 ± 5.9
*Alstroemeria magnifica* var. *sierrae* (4406)	46.0 ± 3.6	51.0 ± 1.8	187.5 ± 7.2
*Alstroemeria magnifica* var. *sierrae* (4407)	47.0 ± 4.2	50.0 ± 2.1	193.5 ± 6.8
*Alstroemeria magnifica* var. *tofoensis* (4409)	55.0 ± 3.9	55.0 ± 1.5	173.9 ± 9.2
*Alstroemeria magnifica* var. *magenta* (4379)	54.0 ± 4.8	51.0 ± 2.4	104.6 ± 8.8
*Alstroemeria magnifica* var. *magenta* (4380)	55.0 ± 5.3	51.0 ± 1.3	100.1 ± 4.6
*Alstroemeria magnifica* var. *magenta* (4381)	55.0 ± 4.4	49.0 ± 1.8	109.4 ± 5.2
*Alstroemeria magnifica* var. *magenta* (4383)	53.0 ± 3.1	50.0 ± 2.2	110.4 ± 5.9

Karyological studies have previously reported about the morphology of chromosomes, patterns and polymorphism of C-bands, nuclear content, and the genomic size of *A. magnifica* (Buitendijk and Ramanna, 1996; Buitendijk *et al.*, 1997, 1998). The present study concurs with the findings made in those publications, specifically on the stability of the 2*n* = 16 present in all varieties of *A. magnifica*. Additionally, our results support the typical asymmetric and bimodal karyotype present in *Alstroemeria*, with four to seven metacentric, submetacentric or subtelocentric chromosomes (Baeza *et al.*, 2008). Within this cytological configuration, it is possible to distinguish, at least, two patterns that can discriminate the varieties of this complex.

First, while identical in structure, notorious differences are noticeable in the total length of chromosomes (TLC). This observation allows to distinguish A. *magnifica* var. *magnifica* from *A. magnifica* var. *sierrae*, as the former exhibits smaller chromosomes than the latter (Table 2), despite exhibiting identical karyotypes (Figures 3A and 3C). Populations from *A. magnifca* var. *sierrae* and *A. magnifica* var. *magnifica* are separated because of their differences in the CV_CL_ index (Figure 5), which is directly related to the TLC values (Perruzzi and Erôglu, 2013). This cytological pattern is intriguing, because of its recurrent presence in other complexes in *Alstroemeria*. For example, in the *Alstroemeria diluta* complex, both recognized subspecies, *A. diluta* subsp. *diluta* and *A. diluta* subsp. *chrysantha*, reveal similar karyotypes but a different TLC values (Baeza *et al.*, 2016a). Such change in chromosome lenght could be the result of changes in the total genomic nuclear size of *A. magnifica* (Buitendijk *et al.*, 1997, 1998), which could have implications as a mechanism of differentiation among taxa in *Alstroemeria*. Nonetheless, this circumstantial evidence should be further corroborated with additional studies based on nuclear DNA content (e.g., flow cytometry) and its variation across natural populations.

The second pattern is exhibited in *A. magnifica* var. *magenta* and *A. magnifica* var. *tofoensis*, which present different and unique karyotypes – compared to *A. magnifica* var. *magnifica* and *A. magnifica* var. *sierrae* (Figures 3 and 4). In this case, chromosome 3 of *A. magnifica* var. *magenta* is submetacentric, instead of subtelocentric or telocentric found in the other varieties of *A. magnifica*. In *A. magnifica* var. *tofoensis*, a polymorphism in the length of chromosome arms is detected between homologous chromosomes of pair 5, which is also expressed in terms of greater levels of magnitude in standard variation related to TCL (Table 2). This pattern is in line with previous reports in *A. philippii*, where a population revealed length polymorphism between homologous in the chromosome pair 3 (Buitendijk *et al.*, 1998). A similar situation has been found in species of *Brachycome*, *Triticum*, *Tulpia*, *Secale*, *Allium* (Houben *et al.*, 2000), *Scilla* (Greilhuber and Speta, 1976), *Placea amoena* (Baeza and Schrader, 2004), and *Chaetanthera pentacaenoides* (Baeza and Torres-Díaz, 2006).

In terms of the overall differentiation among the varieties of the *A. magnifica* complex, a better characterization is possible to achieve by using individual patterns of asymmetry in chromosomes. *A. magnifica* var. *magnifica* and *A. magnifica* var. *magenta* revealed substantial differences in their M_CA_ and CV_CL_ values, which also occurs with *A. magnifica* var. *tofoensis*; nonetheless, the latter with similar patterns than *A. magnifica* var. *magenta* (Figure 5). Despite this, the karyotype of *A. magnifica* var. *magnifica* and *A. magnifica* var. *magenta* exhibits notorious differences, especially in the unique presence of a submetacentric chromosome in pair 3 of *A. magnifica* var. *magenta* compared to the rest of the complex. Furthermore, *A. magnifica* var. *tofoensis* presents a polymorphism in the homologous metacentric chromosomes in pair 5 (see above), while this is subtelocentric in *A. magnifica* var. *magenta*. These unique features suggest that both asymmetry patterns and karyotype variation should be considered together if this evidence is to be used for the precise discrimination of the involved varieties.

The results of this study suggest that patterns of chromosome variation can be instrumental for discriminating among taxa and proposing taxonomic rearrangements in the species complexes of *Alstroemeria*, as they tend to exhibit higher levels of stability and resolution than traditional tepal morphological characters at intraspecific levels (Cajas *et al.*, 2009; Baeza *et al.*, 2010, 2015, 2016a,b). In this case, these changes would be further supported, as cytological data is integrated and contextualized with preliminary results observed from additional character sources (chloroplast DNA, colorimetric variation and morphometry of tepals; Carrasco *et al*. in preparation). For example, given the concordance of cytological data with patterns of discrete morphological variation, sympatric distribution and differentiation based in chloroplast DNA, it is likely that *A. magnifica* var. *sierrae* and *A. magnifica* var. *magnifica* should retain their taxonomic status without modifications. Instead, *A. magnifica* var. *tofoensis* status should be changed to subspecies level, because, despite presenting clear differentiation in floral characters, isolated distribution, and a distinctive unique karyotype, it presents close genetic similarity with *A. magnifica* var. *magnifica* and *A. magnifica* var. *sierrae*. Likewise, it would be recommendable to revalidate *A. magenta* Bayer, from *A. magnifica* var. *magenta*, as originally proposed by Bayer (1987), given its consistent differences in vegetative and floral characters (smaller plants and flowers), allopatric distribution, a distinctive karyotype and substantial genetic distance from the rest of the taxa of *A. magnifica* complex. Nonetheless, these proposals should be seen as tentative, contingent upon additional and more conclusive results that can be added from the suggested character sources.
